# Analysis of Floral Scent and Volatile Profiles of Different *Aster* Species by E-nose and HS-SPME-GC-MS

**DOI:** 10.3390/metabo13040503

**Published:** 2023-03-31

**Authors:** Seung-Yeop Song, Myung-Suk Ahn, Manjulatha Mekapogu, Jae-A Jung, Hyun-Young Song, So-Hyeon Lim, Jong-Sik Jin, Oh-Keun Kwon

**Affiliations:** 1 Floriculture Research Division, National Institute of Horticultural and Herbal Science, Rural Development Administration, Wanju 55365, Jeollabuk-do, Republic of Korea; 2 Department of Oriental Medicine Resources, Jeonbuk National University, 79 Gobong-ro, Iksan 54596, Jeollabuk-do, Republic of Korea

**Keywords:** *Aster*, E-nose, floral scent, GC-MS, secondary metabolites, volatile compounds

## Abstract

Plants from the *Aster* species are known to be a rich source of bioactive chemical compositions and are popularly known for their medicinal properties. To investigate the relationship between the nine species of *Aster*, the floral fragrance and volatile profile patterns were characterized using E-nose and HS-SPME-GC-MS. Initial optimization for fragrance analysis was performed with *Aster yomena* using E-nose by evaluating the scent patterns in different flowering stages. *Aster yomena* exhibited varied scent patterns in each flowering stage, with the highest relative aroma intensity (RAI) in the full flowering stage. PCA analysis to compare and analyze the scent characteristics of nine *Aster* species, showed a species-specific classification. HS-SPME-GC-MS analysis of flowers from nine *Aster* species revealed 52 volatile compounds including β-myrcene, α-phellandrene, D-limonene, trans-β-ocimene, caryophyllene, and β-cadinene. The terpenoid compounds accounted for the largest proportion. Among the nine *Aster* species flowers, *Aster koraiensis* had sesquiterpenes as the major component, and the remaining eight varieties had monoterpenes in abundance. These results could distinguish the species according to the scent patterns and volatile components of the nine *Aster* species. Additionally, flower extracts from the *Aster* species’ plants exhibited radical scavenging antioxidant activity. Among them, it was confirmed that *Aster pseudoglehnii*, *Aster maackii*, and *Aster arenarius* had high antioxidant activity. In conclusion, the results of this study provide fundamental data of the volatile compound properties and antioxidant activity of *Aster* species, offering basic information of valuable natural sources that can be utilized in the pharmaceutical, perfume, and cosmetic industries.

## 1. Introduction

Asteraceae is one of the largest angiosperm families, consisting of 24,000 species, and about 1600 genera that are distributed globally [1]. Asteraceae is characterized by clusters of flowers with the flower head consisting of a ray floret and a disk floret. *Aster* species’ plants belong to the Asteraceae family, and representative *Aster* species’ plants include *Aster scaber*, *Aster tataricus*, *Aster yomena*, *Aster pseudoglehnii*, and *Aster spathulifolius* which are used for ornamental, edible, and medicinal purposes. Since ancient times, plants of the *Aster* species have been used to treat various ailments, including antipyretics, muscle relaxants, and headaches [2]. According to previous studies, *Aster scaber* showed neuroprotective effects, anti-neuroinflammatory activity [3,4], and antioxidant and anti-obesity effects [5]. *Aster tataricus* effectively managed diabetic retinopathy [6], and early detection of contamination and defects in foodstuffs by an electronic nose showed antitussive, expectorant activity [7], anti-inflammatory [8], and antioxidant effects [9]. *Aster yomena* has been shown to have protective effects against acute pancreatitis [10] and various functional studies have been conducted, such as showing antioxidant and anti-inflammatory effects [11]. Experiments were conducted on volatile components of *Aster* species’ plants, such as isolating volatile compounds from *Aster scaber* leaves [12], comparing volatile components of essential oils of *Aster tataricus* and *Aster koraiensis* [13], and searching for volatile components of essential oils extracted from aerial parts of *Aster ageratoides* [14]. However, most studies in the field of *Aster* species have focused on functional effects like antioxidant and anti-inflammatory functions of the plant extract of *Aster* species. Studies on the identification of floral volatile compounds in *Aster* species are limited because most of the species are native to Korea.

Recently, flowers have been used not only for ornamental purposes, but also as industrial raw materials due to the increasing demand for natural compounds in modern medicine, cosmetics, perfumes, and functional food. Volatile compounds in flowers are also widely used in the food, pharmaceutical, and chemical industries [15]. Floral scents emit different types of volatile organic compounds, and play key roles in reproduction by attracting pollinators, and are involved in plant defense, protecting them from pathogens and biohazards [16]. Floral volatiles and their fragrances with biological significance are specific to the developmental stage of the flower and floral organs [17]. The well-known analytical methods for the analysis of volatile components of flowers are the Electronic-nose (E-nose), gas chromatography–flame ionization detector (GC–FID), gas chromatography–mass spectrometer (GC–MS), and gas chromatography–olfactometry (GC–O) [18]. An E-nose is an olfactory simulation test tool that mimics the principle of human olfaction and is an analytical device used for rapidly detecting and identifying volatile organic compounds [19,20]. Most sensor alignment systems can be used to convert chemical signals into electrical signals and then use pattern recognition programs to generate specific odor profiles. Metabolic diseases, freshness of meat, adulteration of milk, and varieties of fruit can be detected with high accuracy using this system [21,22]. Until now, the E-nose has been used in food, perfume, pharmaceutical business, and environmental monitoring [20]. GC–MS is frequently used to analyze volatile compounds in various plants. A study on volatile compounds in plants is being conducted using GC–MS combined with headspace analysis and sample preparation technique (Solid Phase Micro Extraction). Headspace analysis is used to analyze non-volatile liquids and volatile substances in natural materials. SPME is a simple, fast, and sensitive sample preparation technology that allows simultaneous sampling and sample preparation steps and minimizes the use of solvents [23].

As a diagnostic characteristic defining the species related to Asteraceae, the difference in the length of the pappus was identified and distinguished. However, the length of pappus can vary according to the evolutionary process, so it is difficult to subdivide the *Aster* species plants only by their morphological characteristics [24]. Since *Aster* species’ plants have various morphological variations depending on the distribution area [25], it is difficult to distinguish *Aster* species’ plants that have a similar appearance. To distinguish plants with similar appearances, such as *Aster* species’ plants, research is underway to classify plants through molecular biological technology [26], and volatile compounds, as well as genetic characteristics of plants, that can reflect the taxonomic similarity of species [27]. Free radicals including reactive oxygen species (ROS) and reactive nitrogen species (RNS), at an appropriate level help in maintaining the body’s homeostasis, but at higher concentrations they induce oxidative stress, leading to cancer and auto-immune diseases [28]. Synthetic antioxidants like butylated hydroxyanisole (BHA) and butylated hydroxytoulene (BHT) were widely used earlier. However, since they were found to be accompanied with teratogenicity and carcinogenicity, development of effective and safe antioxidants from natural resources is in demand.

Therefore, in this study we profiled the volatile fragrance composition by comparatively analyzing floral fragrance patterns and fragrance components of *Aster* species’ plants using E-nose and HS-SPME-GC-MS and further investigated their antioxidant effects in floral extracts from these plants.

## 2. Materials and Methods

### 2.1. Plant Material

**Figure 1:**
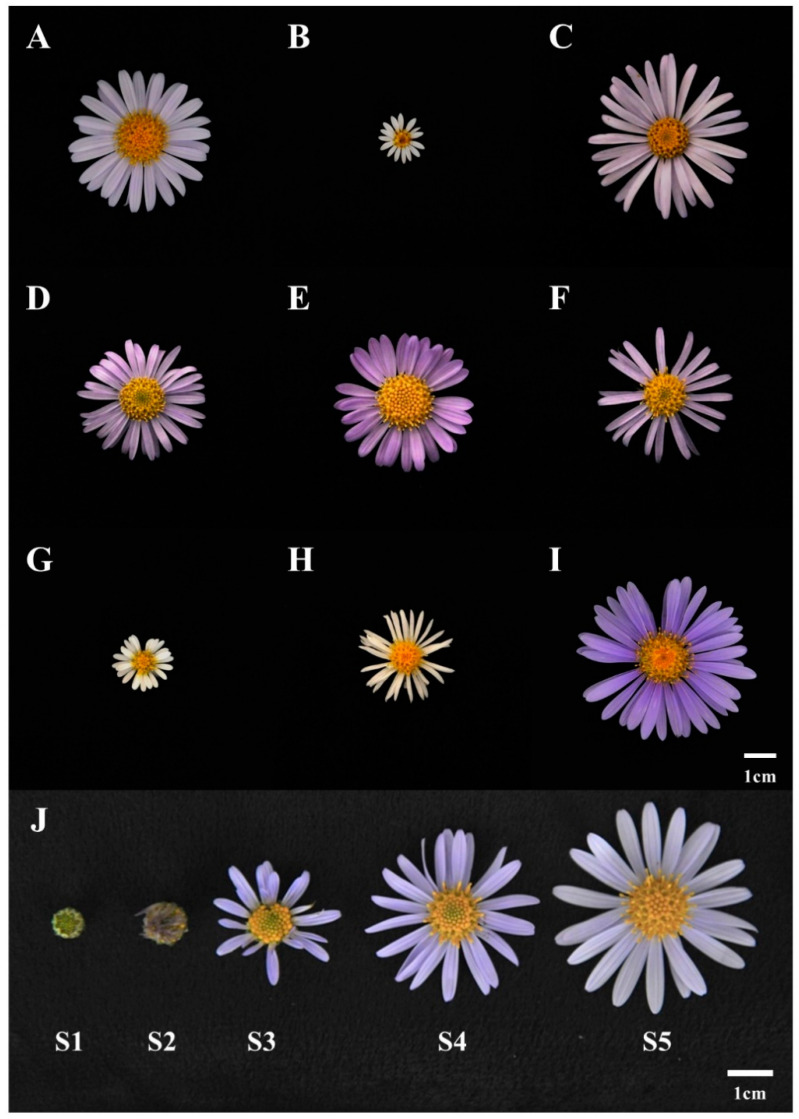
Flower images of nine *Aster* species (**A**) *Aster yomena*; (**B**) *Aster pseudoglehnii*; (**C**) *Aster hispidus*; (**D**) *Aster arenarius*; (**E**) *Aster hayatae*; (**F**) *Aster danyangensis*; (**G**) *Aster pilosus*; (**H**) *Aster maackii*; (**I**) *Aster koraiensis*; (**J**) Different flowering stages of *Aster yomena* (stage 1–5).

A total of nine *Aster* species including *Aster yomena* (AYN), *Aster pseudoglehnii* (APN), *Aster hispidus* (AHD), *Aster arenarius* (AAR), *Aster hayatae* (AHT), *Aster danyangensis* (ADG), *Aster pilosus* (APS), *Aster maackii* (AMK), *Aster koraiensis* (AKS), were used in this study (Figure 1). These *Aster* species were collected from different locations in Korea. For this study, plants of all *Aster* species were raised and maintained in the open field conditions at the National Institute of Horticultural and Herbal Science (Wanju, Korea). Fully opened flowers from about nine plants from each species were used for antioxidant analysis, E-nose and HS-SPME-GC-MS analysis. Flowers were collected between 11:00 a.m. and 03:00 p.m., from August to September during the flowering period in 2021.

### 2.2. Sample Preparation for E-nose Analysis

Different flowering stages of ‘*Aster yomena*’ were initially analyzed to determine the scent pattern and relative aroma intensity (RAI) by E-nose. RAI is a value for comparing scent intensities among the samples. This RAI value represents the Euclidean distance between the centers of samples analyzed in triplicate in the control (air). Three replicates from each sample were used. Various flowering stages used for the study include: (i) Tight bud stage—invisible ray floret and tight buds (S1); (ii) Bud developing stage—initial growth stage of ray floret (S2); (iii) Initial blooming stage—beginning stage of ray floret opening (S3); (iv) Almost opened flower stage—fully bloomed ray floret and initial disk floret opening stage (S4); (v) Fully opened flower stage—fully opened ray and disk florets (S5). About 1 g of the fresh flower sample from all the *Aster* species plants was placed in a 20 mL vial with a screw cap.

### 2.3. Floral Scent Analysis by E-nose

Floral scent was analyzed in nine *Aster* species using an Alpha MOS FOX-2000 E-nose (Alpha MOS, Toulouse, France) containing six different metal oxide sensors. The change in resistance reading rate during vapor exposure of each sensor was analyzed using the chemical sensitivities of six metal oxide semiconductors [29]. The sensor can detect certain compounds (P10/1, P10/2, non-polar volatiles, PA2, T30/1, organic solvents, P40/1, fluorides or chlorides, T70/2, food flavors, and volatile compounds) [30]. P and T are n-type semiconductor sensors. P and T sensor equipped with E-nose (Alpha MOS) are metal oxide sensors based on tin dioxide SnO_2_ (n-type semiconductor), the difference between the sensors lies in the geometry of the sensors. A vial with samples was placed in an automatic sampler (HS-100, CTC Analytics, France). The dry carrier gas flow was set to a constant flow at a rate of 150 mL/min. The vial was then incubated at 40 °C. for 2 min and stirred at 500 rpm (on, 5 s; off, 2 s). Each floral volatile obtained from the headspace was injected into the E-nose sensor chambers at an injection speed of 1000 µL/s. The data acquisition time was 120 s with the default time in between sample injections being 500 s for sensor signals to be at the baseline. E-nose analysis of each sample was performed in three repetitions.

### 2.4. Floral Volatile Component Analysis by HS-SPME-GC-MS

To extract volatile compounds from the fresh flower sample, 2 g of fresh flower sample from all the *Aster* species’ plants was placed in a 20 mL glass vial and sealed with a silicone/PTEE septum. A headspace-solid phase micro extraction (HS-SPME) was carried out using a fiber coated with 80 µm divinylbenzene/carbon wide range/polydimethylsiloxane (DVB/C-WR/PDMS) (PAL SYSTEM, Switzerland) on the automatic HS-SPME autosampler (PAL RSI, PAL System). The conditions of sample incubation were 40 °C and 10 min, and the fiber exposure time was 20 min. The fiber was directly desorbed for 1 min. HP-5MS (Agilent Technologies, Inc., 30 m × 0.25 mm, 0.25 µm) was used as a capillary column and helium (1 mL/min) was used as the carrier gas and the inlet was operated in splitless mode. The oven temperature was initially maintained at 40 °C for 5 min, then raised to 220 °C at the rate of 3 °C/min, and the final temperature of 220 °C was maintained for 5 min. The electron energy was 70 eV, and the mass scan range was 30–500 (3.1 scans/s). NIST 14 (National Institute of Standards and Technology, Gaithersburg, MD, USA) library was used for the mass spectra library, ≤80 for the match factor, and Mass Hunter Qualitative Analysis Workflows (Agilent) for the software. The relative amounts of the volatile compound were calculated using the area of the individual peak relative to the total areas. The results of volatile perfume analysis using HS-SPME-GC-MS were compared with the n-alkane, NIST 14 mass spectrum library to identify compounds, and their retention index (RI) was compared with literature values, and volatile compounds compared to standards were confirmed.

### 2.5. Sample Preparation for DPPH, ABTS Radical Scavenging Assay

Fully opened flowers from about nine plants from each *Aster* species were air-dried. Dried flowers were extracted at room temperature for 3 days with 70% aq. EtOH. After filtration, the extracts were evaporated using a rotary evaporator and freeze-dried.

### 2.6. DPPH Radical Scavenging Assay

DPPH radical scavenging activity was determined using the Prieto method [31]. DPPH was dissolved in MeOH at a concentration of 0.2 mM. After adding the samples and standards to the 96-well microplate, serial dilution was performed according to the sample concentration in the first row of columns 2–12. After covering the plate with a lid to minimize evaporation, it was wrapped with aluminum foil to prevent DPPH radicals from being degraded by light and kept at room temperature for 30 *min*. Sample was then measured at 515 nm using a microplate reader (Agilent Biotek, CA, USA). Assays were run in triplicate for each sample. Ascorbic acid (Vit C) and butylated hydroxyanisole (BHA) were used as standards/positive controls. Assays were run in triplicate for each sample. The results of radical scavenging activity were expressed as IC_50_ values, which represents the concentrations that inhibit DPPH radicals by 50%.

### 2.7. ABTS Radical Scavenging Assay

ABTS radical scavenging activity was determined using the Re, et al. (1999) method [32]. ABTS stock solution was reacted with 2.45 mM potassium persulfate and this mixture was kept in the dark at room temperature for 12–16 h before use to produce the ABTS radical cation. The ABTS solution was diluted with distilled water to an absorbance of 0.70 (±0.02) at 734 nm and equilibrated at 30 °C. ABTS solution was added to the sample, and kept in the dark for 10 min. The absorbance (734 nm) was then read in a microplate reader. All determinations were carried out at least three times, in triplicates and at each separate concentration of the standard and samples. The results were expressed as IC_50_ values, which are concentrations that inhibit ABTS radicals by 50%. Ascorbic acid (VIT C), and butylated hydroxyanisole (BHA) were used as the positive control.

### 2.8. Statistical Analysis

E-nose data was analyzed by multivariate statistical techniques including principal component analysis (PCA) and discriminant function analysis (DFA) using Alpha Soft (V12.45; Alpha MOS, Toulouse, France). Multivariate statistical analysis of GC-MS data was performed using Python, version 2.7.3 (www.python.org (accessed on 10 August 2021)). Hierarchical clustering analysis (HCA) was performed using the R program (version 3.4.0; R core Team, Vienna, Austria) to statistically analyze the comprehensive relationship in each data sample. Heatmapper (www.heatmapper.ca (accessed on 16 January 2023)). was used to visualize the relative content of volatile compounds by cultivar [33]. Antioxidant assay data were analyzed using Graph Pad Prism software, version 5.0 (Graph Pad Software, Inc, La Jolla, CA, USA). One-way analysis of variance using the Tukey post hoc test was used to confirm statistical significance. A value of *p* < 0.05 was considered significant.

## 3. Results

### 3.1. Optimization of Floral Fragrance Pattern Analysis in Flowering Stages of Aster yomena Using E-nose

**Figure 2:**
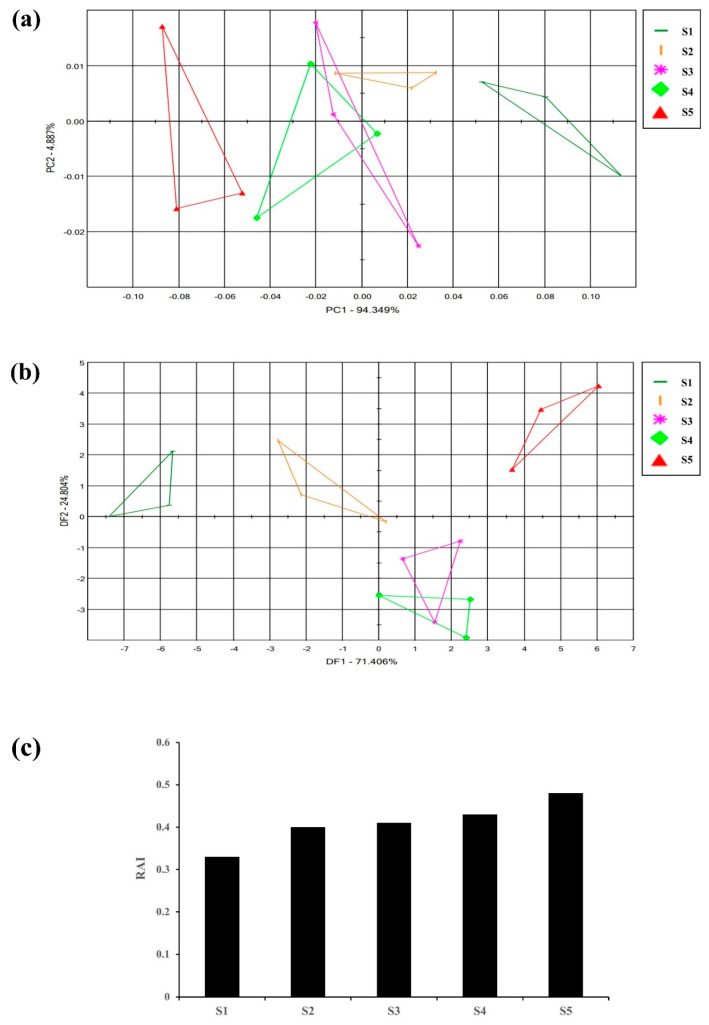
Floral scent patterns of different flowering stages of *Aster yomena* were distinguished by principle component analysis (**a**) discriminant factor analysis; (**b**) graph representing the relative aroma intensity of different flowering stages; (**c**) S1-Tight bud stage, S2-Bud developing stage, S3-Initial blooming stage, S4-Almost opened flower stage, S5-Fully opened flower stage.

Flowers emit different fragrances at various stages of flowering which is species specific [34]. To initially identify the flowering stage suitable for the analysis of the flower scent of the *Aster* species, ‘*Aster yomena*’ was selected among nine *Aster* species to perform E-nose. Multivariate statistical analysis (PCA, DFA) of the E-nose sensor data set and the relative fragrance intensity (RAI) were analyzed. E-nose data for each flowering stage of *Aster yomena* were depicted as a two-dimensional PCA score plot using two significant principal components (PC1 and PC2) that accounted for 94.349% and 4.887% of the total variance of 99.236% (Figure 2a). In the PCA plot, the flower scent of *Aster yomena* exhibited a distinct pattern according to the flowering stage. It was found that the scent pattern S3 and S4 were similar compared to other stages because of their overlapping parts. From these results, it could be confirmed that the characteristics of fragrance varied depending on the stage of flower development.

DFA score plots of the *Aster yomena* E-nose data were plotted as two-dimensional DFA score plots with two principal components (DF1 and DF2) accounting for 71.406% and 24.804% of the total variance of 96.21% (Figure 2b). Relative aroma intensity (RAI) of the E-nose is expressed as the distance between blank (air) and control (sample). A higher RAI value indicates that the aroma intensity of the sample is stronger [34]. The RAI value for each flowering stage was different for each stage (Figure 2c). As the flowering stage progressed, the RAI value increased, and the highest RAI value was 0.48 during the full flowering period. Therefore, based on these results, the standard for flower fragrance analysis of *Aster* species was set as the full flowering stage with the highest relative aroma intensity.

### 3.2. Floral Scent Pattern Analysis of Aster Species Using E-nose

**Figure 3:**
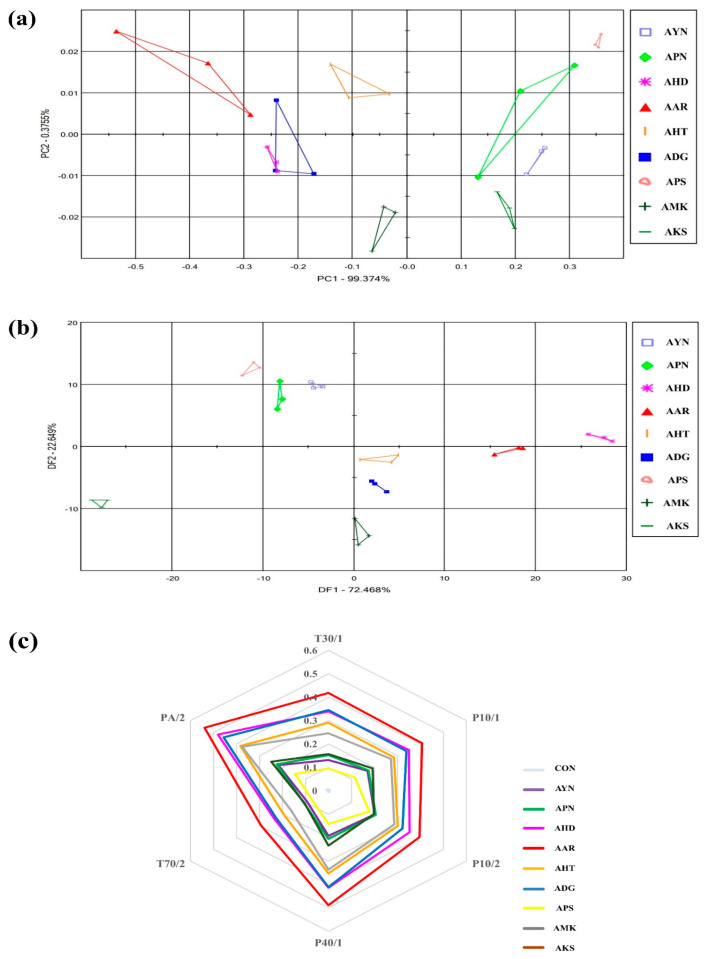
Floral scent patterns of nine *Aster* species are distinguished by principle component analysis (**a**) discriminant factor analysis; (**b**) radar plot representing the discrimination of floral scents of different Aster species by each sensor; (**c**) *Aster yomena* (AYN), *Aster pseudoglehnii* (APN), *Aster hispidus* (AHD), *Aster arenarius* (AAR), *Aster hayatae* (AHT), *Aster danyangensis* (ADG), *Aster pilosus* (APS), *Aster maackii* (AMK), *Aster koraiensis* (AKS).

The flower scent patterns of nine *Aster* species were analyzed in the fully opened flower stage through multivariate statistical analysis of E-nose sensor data. (Figure 3). The E-nose PCA score plot of nine *Aster* species were expressed as a two-dimensional plot using two principal components (PC1, PC2), which showed 99.3% of PC1 and 0.37% of PC2 out of 99.7% of the total variation (Figure 3a). The DFA score plot was expressed as a two-dimensional plot using two principal components DF1 and DF2, and as shown in Figure 3b, DF1 and DF2, showed 72.4% and 22.6%. On the PCA and DFA plots, all samples were located separately according to species. This result indicates that there is a difference in the fragrance pattern depending on the species. Most of the E-nose sensors responded to the cultivars (Figure 3c). The most significant signals were observed in PA/2, P40/1, P10/2, and T30/1. E-nose sensor values were the highest in *Aster arenarius*, and *Aster pilosus* showed lower sensor values.

### 3.3. Evaluation of Volatile Compounds of Aster Species Using HS-SPME-GC-MS Analysis

**Figure 4:**
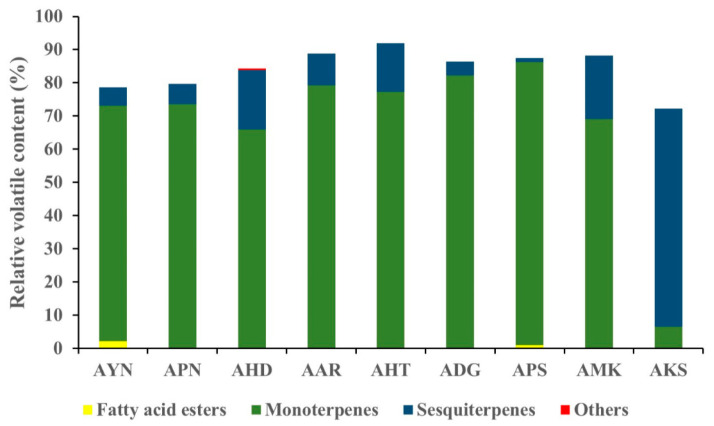
Graph representing the relative content of identified compound classes in the floral volatiles of nine *Aster* species.

**Figure 5:**
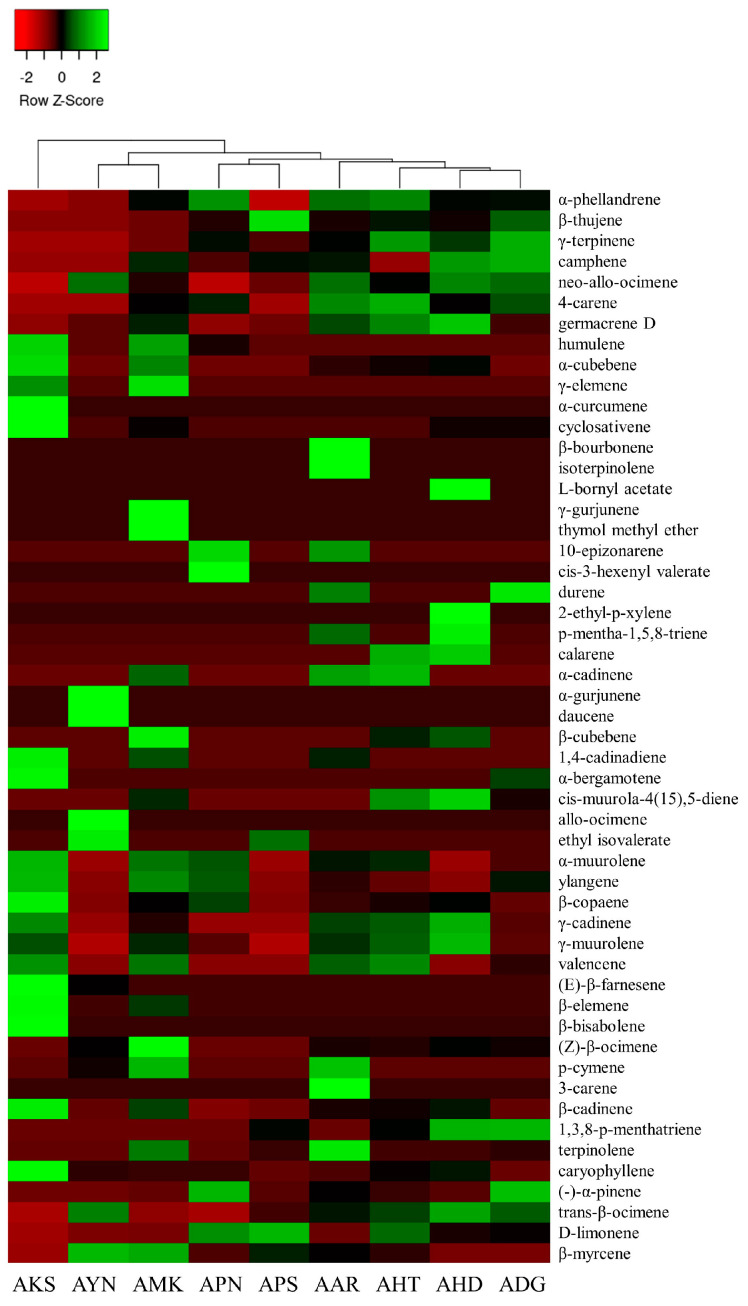
Heat map analysis of the relative content of volatile components in nine *Aster* species. The color scale represents the scaled abundance of each *Aster* species, with green and red colors representing high and low contents, respectively.

Volatile compounds of scent from the flowers of nine *Aster* species were analyzed using HS-SPME-GC-MS. GC-MS analysis detected a total of 52 volatile components in nine *Aster* species’ flowers (Table 1). The identified volatile compounds were categorized as fatty acid esters, monoterpenes, sesquiterpenes, and other compounds according to chemical structures. The terpenoid components predominated in the floral fragrance of all species with the relative content of 72.2–91.9%, accounting for the largest proportion. Among the terpenoid components of nine *Aster* species’ flowers, AKS exhibited the highest relative content of sesquiterpenes (65.6%), and monoterpenes with the relative content of (66.0–85.6%) are the major compounds in the remaining eight species. Fatty acid esters were detected only in AYN, APN, and APS, accounting for 2.2%, 0.3%, and 1%. In the volatile profile the identified compounds accounted for 72.2% (AKS) to 91.9% (AHT) of the total peak area. The total peak area indicates the amount of volatiles present in the species (Figure 4). The relative content of volatile compounds in the *Aster* species was visualized using the heatmapper tool. As a result of the heat map analysis, the scaled abundance of *Aster* species was confirmed for each detected volatile compound (Figure 5).

These results showed that the relative proportions and composition of volatile compounds vary markedly depending on the species of *Aster*.

### 3.4. PCA of Volatile Compounds of Different Aster Species

**Figure 6:**
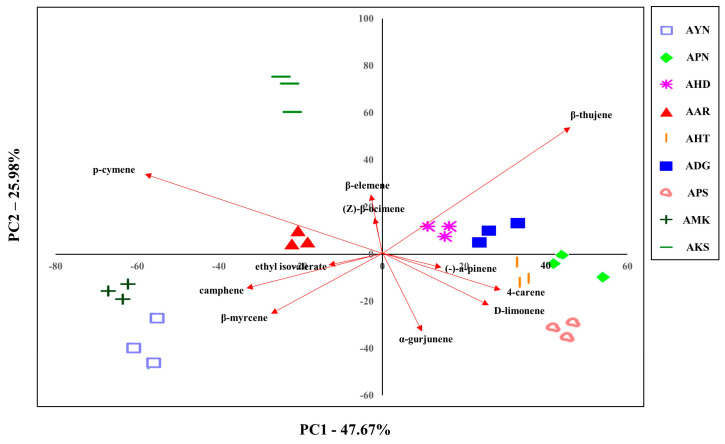
Plot showing the PCA based on the floral volatile compounds of nine *Aster* species’ flowers using GC-MS.

The volatile compounds from the floral scent of nine *Aster* species were evaluated to classify them. Since PCA can be used to identify dataset patterns, volatile compounds obtained by GC-MS were subjected to PCA to compare the pattern and characteristics of floral scents of *Aster* species. The GC-MS PCA score plot is displayed as a two-dimensional plot using two components (PC1, PC2), representing 73.6% of the total variation of the data with PC1 of 47.6% and PC2 of 25.9%. This suggests that PC1 and PC2 are the main components that can differentiate the species (Figure 6). AHD, ADG, APN, AHT, and APS were located in quadrants 1 and 4 based on PC1. AKS, AAR, AMK, and AYN were located in quadrants 2 and 3, and AKS and AYN were the most distant from PC2. These PCA results showed that nine *Aster* species were distinguished according to cultivar.

In addition, the characteristics of the various identified 52 volatile compounds were estimated by PCA. PC1 was composed of the variables like β-thujene, p-cymene, 4-carene, camphene, β-myrcene, and D-limonene compounds. β-thujene, p-cymene, α-gurjunene, β-myrcene, D-limonene, and camphene were the compounds that significantly accounted for PC2. Except for the α-gurjunene, which is a sesquiterpene compound, six of the above compounds are monoterpenes. These PCA results suggested variations in the chemical compositions of the *Aster* species and enabled evaluation of the correlation of their volatile compounds.

### 3.5. Cluster Analysis of Scent Patterns from E-nose and Volatile Compound Compositions from GC-MS

**Figure 7:**
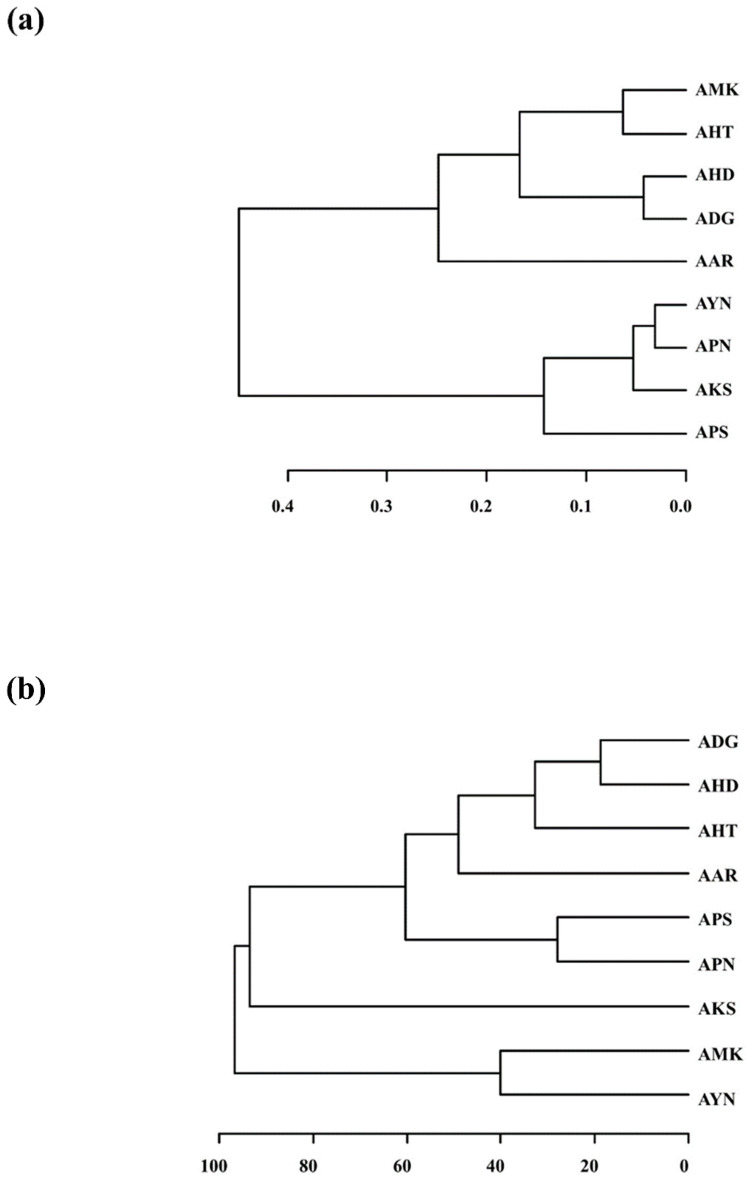
HCA dendrogram depicting the classification of nine *Aster* species from the PCA of E-nose and GC-MS. (**a**) E-nose dendrogram; (**b**) GC-MS dendrogram.

Hierarchical cluster analysis (HCA) based on the E-nose data was performed. The nine *Aster* species formed two major clusters (Figure 7a). Cluster I was divided into two subgroups except for AAR. AMK, AHT, AHD and ADG were grouped into Cluster I, whereas AYN, APN, AKS and APS were grouped into Cluster II. Further, the volatile compound compositions among the nine *Aster* species obtained from GC-MS were compared by performing HCA based on the 52 volatile compounds. The HCA dendrogram based on PCA of GC-MS data indicated that nine *Aster* species formed into two main clusters except for AKS (Figure 7b). Cluster I was divided into two subgroups. Cluster I constituted ADG, AHD, AHT, AAR, APS and APN, whereas Cluster II was formed by AMK and AYN. The cluster analysis results of the E-Nose and the GC-MS indicated that all the nine *Aster* species could be clustered according to their scent patterns and volatile components. AHD, AAR, AHT, and ADG showed a similar clustering pattern. Nevertheless, the branching patterns of the remaining species showed variation. Both PCA and cluster analysis are consistent and could classify all the nine *Aster* species according to their floral scent pattern and volatile compound profile.

### 3.6. Antioxidant Activity of Flowers from Aster Species’ Plants

**Figure 8:**
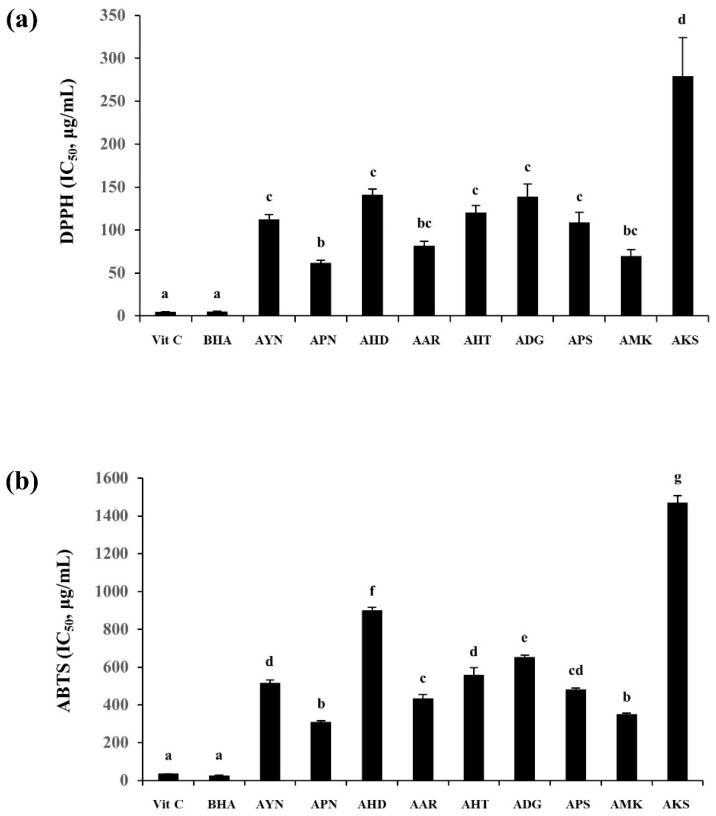
DPPH (**a**) and ABTS (**b**) radical scavenging capacity of the flowers of *Aster* species. Different letters in columns indicate significance from Tukey post hoc test (A value of *p* < 0.05 was considered significant). Error bars indicate standard error of the mean.

DPPH and ABTS radical scavenging activity were expressed using IC_50_ values representing the sample concentration required for 50% inhibition, and ascorbic acid (VIT C), and butylated hydroxyanisole (BHA) were used as the positive control. The highest DPPH radical scavenging capacity among nine *Aster* species, was observed in the flower of APN (IC_50_ = 61.8 ± 2.8 μg/mL) and the lowest was in the AKS flower (IC_50_ = 279.1 ± 44.9 μg/mL). ABTS radical scavenging capacity was also found to be highest in the APN flower (IC_50_ = 310.2 ± 6.9 μg/mL) and lowest in AKS (IC_50_ = 1469.1 ± 38.3 μg/mL) (Figure 8). Correlation analysis of DPPH and ABTS showed a higher correlation between the two assays (R^2^-0.97).

## 4. Discussion

Initial optimization of floral fragrance pattern in different flowering stages of *Aster yomena* showed that the floral scent characteristics varied based on the development of the flowering stage. Since the RAI was highest in the full flowering stage, further flower fragrance analysis by E-nose in the nine *Aster* species was performed in the full flowering stage. Further, floral scent patterns of nine *Aster* species were analyzed in the fully opened flowering stage through multivariate statistical analysis such as PCA and DFA of E-nose sensor data (Figure 3). The PCA and DFA plots of E-nose floral scent of nine *Aster* species showed that all the samples were located separately based on species, which indicates that the scent pattern was species specific. E-nose sensors PA/2, P40/1, P10/2, and T30/1 showed stronger signals. Earlier reports indicated that E-nose sensors including PA/2, P40/1, P10/2, and T30/1 exhibit an affinity for organic solvents, fluoride/chloride, and non-polar volatile substances [30]. Volatile compounds that respond to the mentioned sensors could be candidate compounds that contribute to identifying *Aster* species.

Earlier studies reported that monoterpenes and sesquiterpenes were the main volatile compounds in *Aster scaber* essential oil, and myrcene, limonene, and germanene D were the main volatile compounds [12]. β-myrcene is spicy, woody volatile, and is used as a starting material for commercially important fragrances such as geraniol, linalool, and menthol [35]. It has various therapeutic effects such as antioxidant, anti-inflammatory, and analgesic effects [36,37,38]. D-limonene and trans-β-ocimene were identified in eight species except for AKS. Limonene is a terpene and has a citrus aroma and taste. It is found in citrus oils such as lemon, orange, and grapefruit [39]. It has low toxicity and has been reported to have potential physiological activities including antitumor, antioxidant, and anti-inflammatory effects [40,41]. Trans-β-ocimene has a sweet herbal scent and is one of the wide range of floral volatile organic compounds. It acts as an attractive compound for pollinating species [42,43]. α-pinene is present in seven species except AYN and AKS with higher contents in APN (16.7%) and ADG (17.6%). α-pinene has pine tree aroma and it is also found in high concentrations in *Juniperus oxycedrus* berry and wood oils from cedar of Lebanon. Its flavor is an important starting material for the fragrance industry and it is reported to have antioxidant and hypoglycemic activities [44,45]. Caryophyllene is found in the eight species except ADG, with higher concentration in AKS (24.5%). Caryophyllene is a common, widely present, sesquiterpene anti-inflammatory and has anti-carcinogenic properties [46]. The volatility profile also consisted of key compounds such as β-bisabolene and α-curcumene. These compounds were reported to possess potential anti-cancer properties [47,48]. In addition, terpinolene which is abundant in AAR, is used as a flavoring and synthetic flavoring agent and is also found to have antifungal and antioxidant properties that can prevent LDL oxidation [49,50]. HCA dendrograms based on E-nose and GC-MS data divided the nine *Aster* species into two major clusters (Figure 7a,b). Hence, all the nine *Aster* species could be discriminated according to their scent patterns and volatile components. Previous reports also showed that the cultivars of *Alpinia officinarum* and Chinese *Cybidium* were discriminated based on the differences in volatile components and their characteristics [51,52]. The present study represents the qualitative evaluation of floral volatiles by HS-SPME-GC-MS. This study hence provides basic and useful information to identify potential resources of phytoactive compounds in *Aster* species. However, for the practical application of these compounds, further research is required to evaluate the quantitative implications of the identified candidate compounds.

Among the nine *Aster* species, several plants are known to have antioxidant activity. In a previous study, *Aster yomena* extract had a high DPPH radical, hydroxyl radical, and superoxide radical scavenging ability, confirming its utility as an antioxidant [53]. *Aster pseudoglehnii* extract prevented obesity and oxidative stress when administered to high-fat diet-induced rats [54]. In a study comparing the antioxidant effects of two representative plants of these *Aster* species, it was confirmed that the *Aster pseudoglehnii* extract had better DPPH radical and ABTS radical scavenging activity than the *Aster yomena* extract [55]. In our study, antioxidant analysis of nine *Aster* species using DPPH and ABTS showed that the DPPH radical and ABTS radical scavenging ability of *Aster pseudoglehnii* extract was higher than *Aster yomena*. Antioxidant activity varied in *Aster* species compared to the positive controls VIT C and BHA. This may be because of the fact that, the ethanol extract of the *Aster* species’ flower is crude and constitutes multiple compounds, whereas VIT C and BHA are single compounds. In this study, the correlation analysis between the DPPH and ABTS assay showed a higher correlation (R^2^-0.97). However, since DPPH is a free radical [56] and ABTS is a cation radical [32], the correlation may not be accurate, and a differential activity might be possible because they are based on different free radicals. *Aster arenarius* and *Aster hayatae*, which show similar activities to *Aster yomena* and *Aster pseudoglehnii*, that are known to have antioxidant activity, have not been much studied earlier. These screening results showed the potential of natural antioxidants in *Aster* species. Antioxidant results of this study represent a basic attempt to identify the possible antioxidant sources of *Aster* species. However, further research is required for a deeper understanding of antioxidant effects of identified compounds in detail.

## 5. Conclusions

In conclusion, this study revealed the volatile composition and floral scent patterns of nine *Aster* species using GC-MS and E-nose. The antioxidant effects of flowers from these *Aster* species were analyzed. E-nose analysis revealed species-specific variation in scent patterns and sensors PA/2, P40/1, P10/2, and T30/1 played an important role in distinguishing these scent profiles. GC-MS volatile profiles of *Aster* plants showed that these flowers are rich in phytoactive compounds such as β-myrcene, D-limonene, Trans-β-ocimene, β-bisabolene and caryophyllene. The therapeutic properties of these plants might be related to the abundance of these compounds. Hence, the volatility profile of the *Aster* species plants derived in this study provides a basic source for the medicinal and cosmetic functionalities of these plants. Further research is required to enhance the resolution of exploration of the potential uses of these bioactive compounds and their healthy functionalities.

## Figures and Tables

**Table 1:** Volatile compounds identified, and their contents in nine *Aster* species’ flowers.

No	RI ^a^	Compounds	Relative Content (%) ^b^ ± SD									ID ^d^
			AYN	APN	AHD	AAR	AHT	ADG	APS	AMK	AKS	
		Fatty acid esters										
1	850	ethyl isovalerate	2.28 ± 0.33 ^c^						1.07 ± 0.49			MS, RI
2	1229	cis-3-hexenyl valerate		0.32 ± 0.14								MS, RI
		Monoterpenes										
3	928	(-)-α-pinene		16.77 ± 2.69	1.37 ± 0.22	5.06 ± 1.18	2.66 ± 0.38	17.65 ± 3.15	1.61 ± 0.22	0.83 ± 0.08		MS, RI, STD
4	941	camphene		0.47 ± 0.08	1.86 ± 0.61	0.96 ± 0.17		2.01 ± 0.21	0.92 ± 0.08	1.02 ± 0.09		MS, RI
5	968	β-thujene		2.27 ± 0.32	2.75 ± 0.30	2.62 ± 0.41	3.86 ± 0.20	5.66 ± 0.31	10.66 ± 1.15	0.66 ± 0.07		MS, RI
6	991	β-myrcene	41.46 ± 3.43	13.52 ± 0.52	9.58 ± 0.40	18.98 ± 1.58	15.70 ± 1.11	9.86 ± 1.87	22.67 ± 0.41	38.94 ± 0.48	5.84 ± 0.42	MS, RI, STD
7	998	α-phellandrene	1.09 ± 0.13	5.49 ± 1.76	3.20 ± 0.29	4.83 ± 0.24	5.21 ± 0.22	3.31 ± 0.37		3.21 ± 0.23	0.72 ± 0.31	MS, RI
8	1004	3-carene				4.09 ± 0.63						MS, RI, STD
9	1010	4-carene		1.57 ± 0.29	1.26 ± 0.23	2.59 ± 0.24	3.08 ± 0.15	1.95 ± 0.19		1.25 ± 0.08		MS, RI
10	1019	p-cymene	0.69 ± 0.03			3.78 ± 0.51				3.52 ± 0.06		MS, RI
11	1022	D-limonene	3.66 ± 1.06	31.66 ± 3.52	12.86 ± 1.28	6.14 ± 0.38	26.74 ± 0.89	14.20 ± 1.20	37.93 ± 0.97	4.49 ± 0.25		MS, RI, STD
12	1034	(Z)-β-ocimene	0.70 ± 0.23		0.74 ± 0.06	0.53 ± 0.03	0.44 ± 0.05	0.59 ± 0.16		3.55 ± 0.12		MS, RI
13	1047	trans-β-ocimene	19.41 ± 4.26	0.79 ± 0.24	23.04 ± 0.31	12.43 ± 0.75	14.67 ± 2.20	16.45 ± 3.81	7.47 ± 0.65	2.41 ± 0.20		MS, RI
14	1052	γ-terpinene		0.77 ± 0.04	0.91 ± 0.08	0.70 ± 0.05	1.46 ± 0.07	1.60 ± 0.07	0.42 ± 0.05	0.25 ± 0.03		MS, RI
15	1065	isoterpinolene				0.12 ± 0.02						MS, RI
16	1083	terpinolene			0.91 ± 0.11	14.74 ± 1.53	0.90 ± 0.01	1.63 ± 0.09	1.24 ± 0.11	8.08 ± 0.61		MS, RI, STD
17	1101	durene				0.15 ± 0.00		0.27 ± 0.06				MS, RI
18	1114	p-mentha-1,5,8-triene			0.32 ± 0.04	0.14 ± 0.01						MS, RI
19	1122	allo-ocimene	2.50 ± 0.47									MS, RI
20	1123	1,3,8-p-menthatriene			5.59 ± 0.35		1.70 ± 0.48	5.74 ± 1.22	1.88 ± 0.13			MS, RI
21	1134	neo-allo-ocimene	1.35 ± 0.30		1.48 ± 0.12	1.35 ± 0.06	0.87 ± 0.15	1.33 ± 0.28	0.46 ± 0.03	0.71 ± 0.08		MS, RI
22	1227	thymol methyl ether								0.23 ± 0.05		MS, RI
23	1276	L-bornyl acetate			0.13 ± 0.05							MS, RI
		Sesquiterpenes										
24	1340	α-cubebene			0.40 ± 0.03	0.22 ± 0.06	0.29 ± 0.09			0.94 ± 0.07	1.47 ± 0.32	MS, RI
25	1357	cyclosativene			0.21 ± 0.01			0.21 ± 0.06		0.27 ± 0.04	2.08 ± 0.77	MS, RI
26	1366	ylangene		0.98 ± 0.36		0.42 ± 0.08	0.22 ± 0.07	0.70 ± 0.20		1.29 ± 0.04	1.66 ± 0.43	MS, RI
27	1371	daucene	0.64 ± 0.44									MS, RI
28	1375	β-bourbonene				0.14 ± 0.03						MS, RI
29	1381	β-cubebene			0.27 ± 0.01		0.19 ± 0.06			0.69 ± 0.02		MS, RI
30	1384	β-elemene								1.22 ± 0.10	4.38 ± 1.51	MS, RI
31	1400	α-gurjunene	0.59 ± 0.41									MS, RI
32	1410	caryophyllene	2.59 ± 1.68	2.39 ± 0.50	6.25 ± 0.33	1.45 ± 0.29	4.44 ± 1.04		0.34 ± 0.06	2.25 ± 0.11	24.54 ± 5.05	MS, RI, STD
33	1420	β-copaene		1.03 ± 0.51	0.69 ± 0.03	0.39 ± 0.13	0.52 ± 0.18	0.20 ± 0.05		0.66 ± 0.06	2.53 ± 0.22	MS, RI
34	1427	γ-elemene								2.18 ± 0.16	1.38 ± 0.67	MS, RI
35	1429	α-bergamotene						0.27 ± 0.02			0.92 ± 0.14	MS, RI
36	1430	calarene			0.21 ± 0.01		0.18 ± 0.05					MS, RI
37	1445	humulene		0.20 ± 0.12						1.05 ± 0.06	1.39 ± 0.36	MS, RI
38	1452	(E)-β-farnesene	0.51 ± 0.34								4.31 ± 0.96	MS, RI
39	1455	cis-muurola-4(15),5-diene			0.90 ± 0.03		0.65 ± 0.26	0.17 ± 0.04		0.32 ± 0.04		MS, RI
40	1471	γ-muurolene		0.40 ± 0.21	1.54 ± 0.27	0.84 ± 0.26	1.03 ± 0.35	0.36 ± 0.09		0.79 ± 0.07	0.93 ± 0.19	MS, RI
41	1474	germacrene D	0.55 ± 0.38		3.82 ± 0.39	1.97 ± 0.63	2.76 ± 0.86	0.78 ± 0.21	0.39 ± 0.09	1.68 ± 0.15		MS, RI
42	1477	α-curcumene									2.04 ± 0.55	MS, RI
43	1479	γ-gurjunene								0.19 ± 0.02		MS, RI
44	1488	valencene				0.72 ± 0.24	0.89 ± 0.32	0.28 ± 0.07		0.80 ± 0.06	0.95 ± 0.21	MS, RI
45	1494	α-muurolene		0.87 ± 0.43		0.67 ± 0.22	0.71 ± 0.24	0.33 ± 0.08		1.05 ± 0.05	1.45 ± 0.23	MS, RI
46	1501	10-epizonarene		0.23 ± 0.12		0.16 ± 0.06						MS, RI
47	1504	β-bisabolene									7.06 ± 1.75	MS, RI
48	1508	γ-cadinene			1.27 ± 0.19	0.71 ± 0.23	0.83 ± 0.29	0.27 ± 0.07		0.40 ± 0.00	1.05 ± 0.20	MS, RI
49	1519	β-cadinene	0.58 ± 0.34		2.26 ± 0.24	1.42 ± 0.41	1.55 ± 0.53	0.56 ± 0.14	0.33 ± 0.07	2.82 ± 0.06	6.82 ± 2.17	MS, RI
50	1527	1,4-cadinadiene				0.19 ± 0.06				0.27 ± 0.01	0.74 ± 0.12	MS, RI
51	1532	α-cadinene				0.28 ± 0.10	0.32 ± 0.12			0.19 ± 0.01		MS, RI
		Others										
52	1074	2-ethyl-p-xylene			0.48 ± 0.05							MS, RI
			78.60	79.73	84.31	88.80	91.90	86.38	87.39	88.18	72.24	

^a^ Retention indices were calculated against n-alkanes (C7-C40) on the HP-5MS column. ^b^ Relative contents (%) = (area under peak/total peak area) × 100. The peak areas were obtained by the total ion chromatographic (TIC) analysis. ^c^ All data are presented as mean standard deviation (*n* = 3). ^d^ MS = by comparison of the mass spectrum with the NIST library, RI = by comparison of RI (retention index) with RI of published literatures and online library (https://webbook.nist.gov/chemistry/cas-ser.html (accessed on 15 April 2021)), STD = by comparison of retention time and mass spectrum of the authentic stand.

## Data Availability

All the data is contained within the article.
